# Life-saving ECMO and fiberoptic bronchoscope thrombectomy for severe respiratory dysfunction in pregnancy: a case report

**DOI:** 10.3389/fmed.2025.1526880

**Published:** 2025-03-17

**Authors:** Chiwen Liu, Li Jiang, Donglan Yuan, Xinlan Xu, Jing Wei

**Affiliations:** Department of Obstetrics and Gynecology, The Affiliated Taizhou People’s Hospital of Nanjing Medical University (Taizhou People’s Hospital), Taizhou, China

**Keywords:** bronchial artery embolization, ECMO, fiberoptic bronchoscope, respiratory dysfunction, pregnancy

## Abstract

**Background:**

Severe respiratory dysfunction during pregnancy, though rare, represents a life-threatening condition, often presenting as dyspnea and respiratory distress. Pregnant patients with pulmonary vascular disease are particularly vulnerable, facing a poor prognosis and a heightened risk of mortality. This report aimed to highlight strategies for mitigating severe complications in high-risk pregnant women and to provide valuable insights into effective clinical management approaches.

**Case presentation:**

We presented the case of a 40-year-old pregnant woman who required hospitalization for intensive monitoring of vital signs. On admission, her temperature was 36.2°C, respiratory rate 25 breaths per minute, blood pressure 108/84 mmHg, and heart rate 87 beats per minute. Notably, her resting blood oxygen saturation was critically low at 80%. A bedside chest X-ray revealed right lung atelectasis with increased interstitial markings and thickening in the left lung. Computed tomographic angiography (CTA) of the thoracic aorta demonstrated a mildly dilated and tortuous bronchial artery supplying the right lung. The patient subsequently developed pulmonary hemorrhage, atelectasis, and pulmonary infection, ultimately progressing to respiratory failure due to congenital bronchial artery malformation. A multidisciplinary intervention strategy was implemented, incorporating extracorporeal membrane oxygenation (ECMO), bronchial artery embolization, fiberoptic bronchoscopic suctioning, alveolar lavage, and comprehensive life support measures. ECMO combined with fiberoptic bronchoscope thrombectomy proved to be instrumental in stabilizing her condition, leading to significant clinical improvement and a successful discharge.

**Conclusion:**

Pulmonary vascular disease-induced hemodynamic instability imposed a substantial risk of circulatory shock in pregnancy. This case underscored the efficacy of ECMO and fiberoptic bronchoscope thrombectomy in the management of severe respiratory dysfunction during pregnancy, advocating for their integration into clinical practice for similar high-risk cases.

## Introduction

We presented the case of a pregnant woman with a history of pulmonary vascular anomalies who suffered recurrent hemoptysis during her pregnancy. At 37 weeks of gestation, her condition rapidly deteriorated, culminating in persistent hypoxemia, respiratory failure, and other severe respiratory dysfunctions. This case posed numerous clinical challenges and dilemmas, demanding a sophisticated and multidisciplinary approach to her care.

## Case report

The patient, a 40-year-old woman in her second pregnancy, had a history of a cesarean section performed in 2011 and a laparoscopic ovarian cystectomy in 2022. In 2013, she was admitted to Jingjiang People’s Hospital due to hemoptysis. At that time, a chest CT scan revealed a lesion in the right lower lobe of her lung, and a computed tomography angiography (CTA) showed an independent bronchial artery at the upper margin of the T6 vertebra with mild thickening and tortuosity, suggesting a bronchial artery malformation. Notably, the patient had remained free from hemoptysis for the past decade. No significant family medical history was reported.

Her last menstrual period was on May 26, 2023, with an estimated delivery date of March 3, 2024. At 26 weeks of gestation, the patient underwent an oral glucose tolerance test (OGTT) during a routine prenatal examination, which confirmed a diagnosis of gestational diabetes mellitus (GDM). The condition was effectively managed through dietary modifications. No evidence of gestational hypertension (GH) was observed. At 5 months of gestation, she experienced intermittent hemoptysis with a small amount of blood and was admitted to Jingjiang People’s Hospital’s respiratory department for observation. After improving within 3–4 days, she was discharged. Throughout her pregnancy, she had four episodes of intermittent hemoptysis but did not require hospitalization for treatment.

On February 9, 2024, at 4 PM, she experienced intermittent hemoptysis four times, totaling approximately 100 mL of blood, and urgently sought care at an outside hospital. Phentolamine and carbazochrome were administered via intravenous infusion to stop the bleeding, along with antibiotics for infection control. Her resting blood oxygen saturation was measured at 80%, fluctuating between 89 and 91% with supplemental oxygen via face mask. There were no signs of abdominal distension or pain, vaginal bleeding, or discharge. The patient was transferred to our hospital at 2:11 PM on February 11, 2024.

The patient was admitted for vital sign monitoring, presenting with a temperature of 36.2°C, respirations of 25 per minute, normal blood pressure, and a heart rate within the normal range. Upon physical examination, decreased breath sounds were noted in the left upper lobe, and a distended abdomen was observed without tenderness or rebound pain. The fetal heart rate was 145 beats per minute, and oxygen saturation was low.

Following a multidisciplinary discussion, an emergency cesarean section under general anesthesia was performed at 15:00 on February 11, with the patient in the supine position. A mature female infant, with Apgar scores of 8 and 9 at 1 and 5 min, respectively, was delivered and subsequently transferred to the neonatal intensive care unit (NICU). During the surgery, the patient’s oxygen saturation remained stable at 99% with endotracheal intubation.

**Figure 1:**
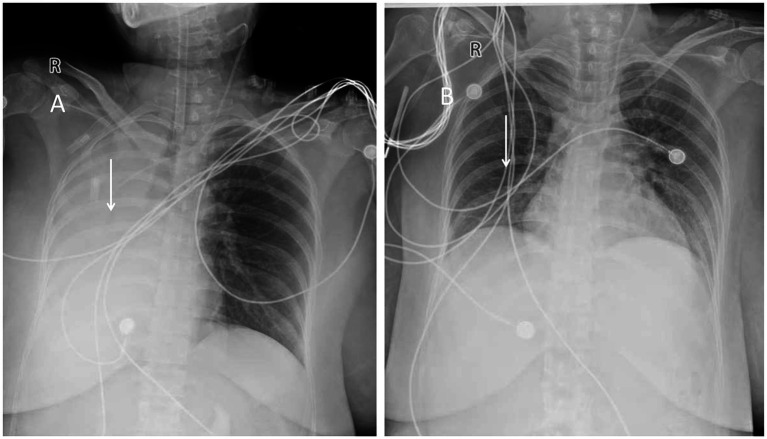
**(A)** Chest X-ray showing right lung atelectasis. **(B)** Follow-up chest X-ray showing near-normal appearance.

Post-operation, the patient was transferred to the intensive care unit (ICU) with endotracheal intubation and assisted ventilation using a ventilator. She received piperacillin-tazobactam antibiotics, expectorants, hemostasis measures, analgesia, and electrolyte balance maintenance, among other symptomatic treatments. A bedside chest X-ray revealed right atelectasis and increased left lung interstitial markings and thickening (Figure 1A). CTA of the pulmonary vasculature showed left upper lobe atelectasis and diffuse inflammation in both lungs progressing from the current film. The CTA of the thoracic aorta demonstrated a mildly dilated and tortuous bronchial artery supplying the right lung, originating from the right side of the thoracic aorta at approximately the T6 vertebral level.

**Figure 2:**
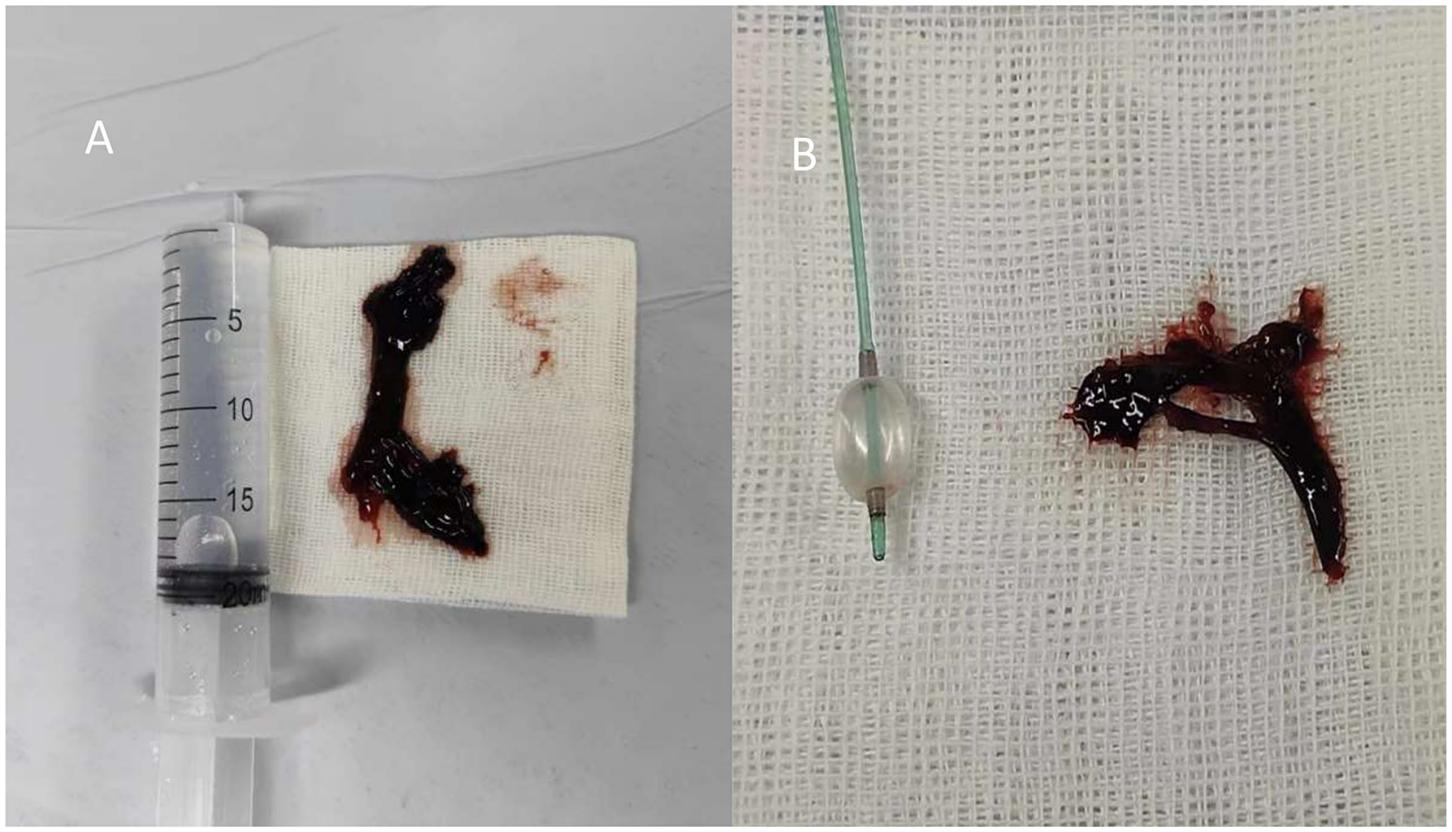
**(A)** Bronchoscopy for thrombus removal. **(B)** Balloon and forceps grasping for thrombus removal.

Despite adjustments to ventilator settings, sedation, and analgesia, the patient’s oxygen saturation remained critically low, fluctuating between 65 and 76%. She continued to experience chest tightness and shortness of breath. On February 11 at 22:40, after further multidisciplinary discussion, extracorporeal membrane oxygenation (ECMO) therapy was initiated. The patient, with a history of bronchial artery malformation and hemoptysis and evidence of atelectasis on chest X-ray, was suspected to have airway obstruction due to blood clots. Following another multidisciplinary discussion, the patient underwent anesthesia and fiberoptic laryngoscope intubation at 02:19 on February 12. Hemorrhagic sputum was aspirated through the tube, and a pulmonary lavage was performed using a fiberoptic bronchoscope at 03:50 while maintaining the patient’s blood oxygen saturation at 88–100% (Figure 2A).

**Figure 3:**
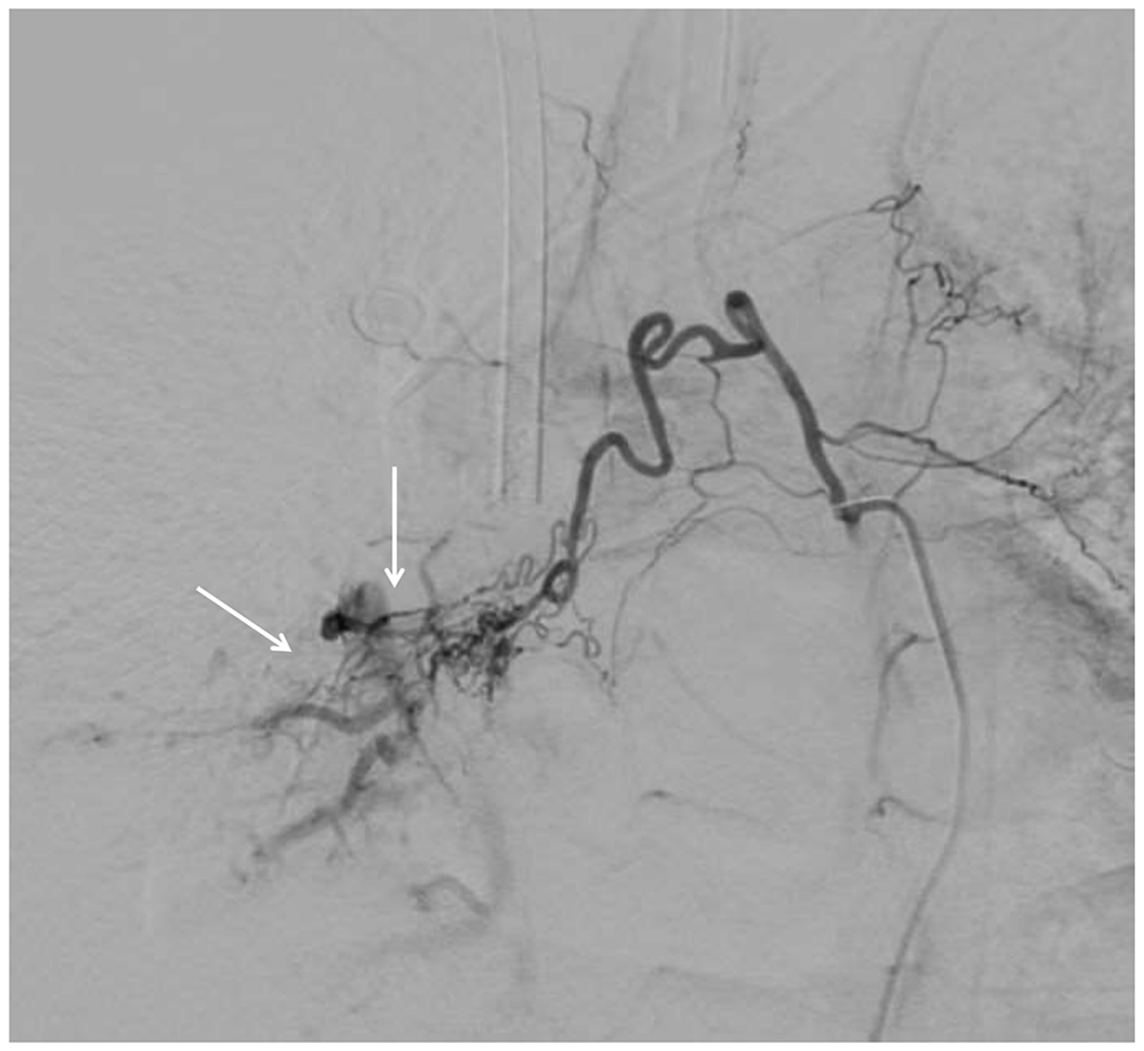
DSA showing contrast agent extravasation (indicated by the arrow).

Despite the intervention, the patient’s vital signs remained unstable, urgently necessitating attention to hemoptysis and atelectasis. Consequently, the interventional radiology team was consulted for an emergency bronchial artery embolization, which was performed at 11:00 AM on February 12 (Figure 3).

During the procedure, a tortuous branch of the right subclavian artery at the T6 level was found to supply the right lung, with evident extravasation of contrast agent in the distal right bronchial artery. The T5 level subcostal angiogram of the right bronchial artery showed a marked exudate sign at the distal end, indicating the presence of a vascular fistula. A catheter was used to deliver a mixture of embolic microsphere particles and iodixanol injection. The follow-up angiogram revealed residual staining of the main trunk of the right bronchial artery without any signs of exudate or distal vessel visualization.

On February 12, at 14:50, a fiberoptic bronchoscope was used for sputum suction and pulmonary alveolar lavage. This revealed substantial blood clots obstructing the opening of the right main bronchus and the branch bronchi of the right upper, middle, and lower lobes. A novel technique involving the use of a balloon and alternating forceps grasping was introduced to address this problem (Figure 2B). Further inspection of the right lower lobe bronchus revealed more blood clots and active bleeding in the bronchial mucosa. The bleeding was effectively managed with repeated administrations of a diluted adrenaline solution. Throughout the procedure, oxygen saturation levels were maintained between 95 and 100%. A bedside chest X-ray taken at 15:53 on the same day showed a significant improvement in lung expansion compared to the previous day.

ECMO treatment was discontinued on February 13. However, on February 14, the patient experienced a recurrent fever, reaching a peak temperature of 38.2°C, prompting a switch to Biapenem antibiotics. Despite this complication, oxygen saturation levels remained stable at 99–100%, blood pressure returned to normal, and subsequent chest X-rays indicated significant re-expansion of the right lung (Figure 1B), along with reduced inflammation in both lungs compared to previous assessments. Following the removal of the tracheal tube, the patient was able to expectorate dark red blood-tinged sputum.

On February 16, a chest CT scan showed scattered inflammations in both lungs with underexpansion observed in the lower lobes. After receiving symptomatic treatment, the patient’s condition stabilized, and she was transferred to the general ward, where she stayed for 8 days before being discharged upon making a full recovery. There were no significant issues noted at a follow-up visit 1 week after discharge. A CT scan performed 3 months post-surgery showed no abnormalities in either lung. During a follow-up evaluation 1 year after the procedure, the patient remained asymptomatic, with no recurrence of hemoptysis, cough, chest tightness, asthma, or fever.

## Discussion

Throughout pregnancy, the respiratory system undergoes significant functional changes, including increased ventilation, elevated alveolar ventilation, hyperventilation, and heightened oxygen consumption. These physiological adjustments are accompanied by uterine enlargement, diaphragm elevation, thoracic diameter expansion, and respiratory mucosa edema and congestion. Consequently, the respiratory defense mechanisms are weakened, leading to an increased susceptibility to lung disease and respiratory dysfunction.

Respiratory dysfunction in pregnancy can manifest as pneumonia, pulmonary vascular disease, and heart failure, with severe cases potentially resulting in respiratory and circulatory failure, septic shock, and multiple organ dysfunction syndrome. These complications pose serious risks for both the mother and fetus, leading to adverse outcomes such as premature birth and fetal distress.

Pregnancy-associated pulmonary vascular diseases encompass a range of conditions, including pulmonary embolism, pulmonary hypertension, pulmonary vasculitis, primary pulmonary artery tumors, pulmonary aneurysmal dissection, and the clinically rare but high-mortality rate pulmonary arteriovenous malformations, often accompanied by severe complications such as diffuse alveolar hemorrhage and pulmonary capillary involvement (1).

A chest CT scan can accurately depict the size and extent of lung lesions, typically presenting as round or oval nodules with uniform density and clear borders, often with visible supply vessels. CT pulmonary angiography (CTPA) and nuclear medicine pulmonary imaging or ventilation/perfusion (V/Q) scans are primary diagnostic tools for pulmonary vascular disease. The combination of a high D-dimer value (>1,000) significantly aids in the prompt diagnosis of pulmonary embolism (2, 3).

Patients may present with symptoms such as chest tightness, dyspnea, cough, hemoptysis, and persistent hypoxemia. If pulmonary hypertension is suspected, echocardiography is routinely performed. In cases of low oxygen saturation during pregnancy, pulmonary arteriovenous shunting should be considered, and right heart acoustic angiography has shown high sensitivity for detecting pulmonary arteriovenous right-to-left shunting (4).

For pregnant women presenting with severe symptoms such as hemoptysis, hypoxemia, and dyspnea early in pregnancy, screening for pulmonary arteriovenous malformation is advisable, particularly if there is a family history of the condition. Embolization is a commonly used and effective treatment modality. Research has demonstrated that embolization for pulmonary arteriovenous malformation at 16 weeks of gestation is both safe and effective. Women experiencing severe pulmonary arteriovenous malformations during the middle and late stages of pregnancy can also consider embolization therapy (5).

In this case, the patient had undergone a cesarean section 14 years prior. Approximately 1 year after delivery, she experienced an episode of sudden hemoptysis without any apparent trigger. She had a 10-year history of bronchial artery malformation but had not exhibited hemoptysis before her current pregnancy. However, hemoptysis began intermittently at 20 weeks of gestation, occurring five times. Symptomatic treatment was administered, but no imaging examinations were performed during pregnancy.

At 37 weeks of gestation, hemoptysis recurred, and conservative treatment was attempted at a local respiratory department, but with limited effectiveness. Upon transfer to our hospital, the patient presented with chest tightness and dyspnea. Critical signs included oxygen saturation fluctuating between 72 and 90% despite ventilator-assisted pure oxygen support, hemoglobin levels of 80 g/L, and recurrent fever following cesarean section. Additionally, pink frothy sputum was observed in the tracheal tube, further indicating respiratory distress.

A CT scan revealed the presence of a pulmonary artery malformation. During surgery, the cause of hemoptysis was confirmed to be a bronchial-venous malformation. Had the patient undergone bronchial artery embolization before or during pregnancy, the outcome might have been more favorable. This highlighted the importance of early intervention and monitoring in patients with known pulmonary vascular anomalies during pregnancy.

ECMO stands at the forefront of advanced life support technologies, providing critical cardiac and respiratory support in diverse modes. This remarkable intervention has proven capable of rescuing patients from the brink of heart and respiratory failure, dramatically reducing the three-month mortality rate. Its adoption in ICU settings is on the rise, reflecting its life-saving potential (6). ECMO is also emerging as a promising therapeutic option in other clinical scenarios, particularly for patients with traumatic injuries and severe burns (7). However, the application of ECMO in pregnant women remains rare, primarily due to the elevated risks of maternal and fetal bleeding, hemolysis, and thrombosis associated with mechanical trauma and anticoagulant therapy. The absence of definitive guidelines or conclusive literature addressing the safety and efficacy of ECMO during pregnancy and the postpartum period further complicates its use in these delicate situations.

While instances of mortality following ECMO usage have been reported, recent advancements in ECMO technology, coupled with pharmaceutical interventions, have significantly enhanced gas exchange and vital organ function, effectively acting as a bridge to restore cardiopulmonary function (8). Moreover, there have been successful cases of women with pulmonary hypertension undergoing cesarean section deliveries with ECMO support, resulting in the survival of both mother and child (9). In our country, a notable case involved a pregnant woman with acute fatty liver disease, which led to coagulation disorders. Following a cesarean section, she developed respiratory distress and persistent hypoxemia and was later diagnosed with pulmonary hemorrhage. Multidisciplinary treatment, including the use of ECMO, led to her successful recovery, offering valuable insights for future clinical practice (10). A 9-year, single-center study has reported by ACOG examined 54 patients with cardiopulmonary failure who have received ECMO. Among them, nine are pregnant or within 6 weeks postpartum, with a maternal survival rate of 33% and a neonatal survival rate of 60%. In contrast, among the 45 non-pregnant patients, 53% (23 cases) survive with ECMO support. The findings suggest that early initiation of ECMO in pregnant women with cardiopulmonary failure may improve maternal survival rates (11).

In this instance, the patient’s pre-operative chest X-ray revealed right lung atelectasis accompanied by scattered inflammation and bronchial obstruction caused by blood clots. The patient underwent two successful bronchoscopic thrombectomies and a bronchial artery embolization, all while receiving ECMO support. This advanced life-sustaining assistance enabled timely and effective intervention, ensuring both immediate and long-term patient stability.

The use of ECMO requires anticoagulation therapy to prevent thrombus formation within the circuit, which in turn increases the risk of bleeding complications, including intracranial hemorrhage, gastrointestinal bleeding, and surgical site hemorrhage. Despite anticoagulation measures, thrombotic events may still occur during ECMO management, potentially leading to severe complications such as pulmonary and cerebral embolism.

Recent evidence suggests that blood exposure to the ECMO circuit can activate the immune system, triggering an inflammatory response and contributing to systemic dysfunction (12). Additionally, ECMO use has been associated with an increased risk of pulmonary and bloodstream infections. However, in this case, the successful application of ECMO highlighted its potential as a life-saving intervention for pregnant women experiencing severe respiratory and cardiac failure. Although the risks associated with ECMO are considerable, its benefits in critical situations can outweigh these risks, particularly with advancements in technology and multidisciplinary care. This case contributed to the growing body of evidence supporting the judicious use of ECMO in pregnancy, highlighting the need for further research and the development of guidelines to ensure the safety and efficacy of this technology in maternal care.

In pregnant patients presenting with symptoms such as hemoptysis or dyspnea, pulmonary vascular diseases should be considered as a potential underlying cause. Early evaluation is crucial for pregnant women who may require ECMO support. The involvement of these departments was warranted due to the intricate relationship between the trachea and blood vessels in thrombus and blood clot removal. Each specialty, interventional, vascular, and neurosurgery, brought unique expertise and employed specialized instruments. The surgical techniques utilized were complementary, with approaches such as pulmonary embolism management, fiberoptic bronchoscopy, and balloon catheterization working synergistically to facilitate the effective removal of blood clots from both the vasculature and bronchi, ensuring a multidisciplinary and comprehensive treatment strategy.

Generally, ECMO initiation should be considered when there is progressive clinical deterioration despite 12–24 h of intensive conventional therapy, including adequate oxygen supplementation, mechanical ventilation, and pharmacological management. However, in cases where the disease progresses rapidly or conventional treatments fail to provide timely intervention, particularly in instances of severe hypoxemia and hemodynamic instability, ECMO should be promptly assessed and implemented as early as possible, even if the standard duration for conventional treatment has not yet been met. Crafting a customized diagnostic and treatment plan tailored to the specific condition of the pregnant woman is essential to ensure the utmost safety of both mother and fetus.

## Data Availability

The original contributions presented in the study are included in the article/supplementary material, further inquiries can be directed to the corresponding authors.
